# Individual honey bee (*Apis cerana*) foragers adjust their fuel load to match variability in forage reward

**DOI:** 10.1038/srep16418

**Published:** 2015-11-09

**Authors:** Ken Tan, Tanya Latty, Shihao Dong, Xiwen Liu, Chao Wang, Benjamin P. Oldroyd

**Affiliations:** 1 Key Laboratory of Tropical Forest Ecology, Xishuangbanna Tropical Botanic Garden, Chinese Academy of Science, Kunming, Yunnan Province, People’s Republic of China; 2 Eastern Bee Research Institute, of Yunnan Agricultural University, Heilongtan, Kunming, People’s Republic of China; 3 Faculty of Agriculture and Environment, Australian Technology Park, University of Sydney, NSW 2015, Australia; 4 Behavior and Genetics of Social Insects Laboratory, Macleay Building A12, School of Biological Sciences, University of Sydney, NSW 2006, Australia

## Abstract

Animals may adjust their behavior according to their perception of risk. Here we show that free-flying honey bee (*Apis cerana*) foragers mitigate the risk of starvation in the field when foraging on a food source that offers variable rewards by carrying more ‘fuel’ food on their outward journey. We trained foragers to a feeder located 1.2 km from each of four colonies. On average foragers carried 12.7% greater volume of fuel, equivalent to 30.2% more glucose when foraging on a variable source (a random sequence of 0.5, 1.5 and 2.5 M sucrose solution, average sucrose content 1.5 M) than when forging on a consistent source (constant 1.5 M sucrose solution). Our findings complement an earlier study that showed that foragers decrease their fuel load as they become more familiar with a foraging place. We suggest that honey bee foragers are risk sensitive, and carry more fuel to minimize the risk of starvation in the field when a foraging trip is perceived as being risky, either because the forager is unfamiliar with the foraging site, or because the forage available at a familiar site offers variable rewards.

Many animals live in environments where the quality or quantity of food resources can change suddenly and without warning. According to risk sensitive foraging theory, animals forage in ways that not only maximize net caloric intake, but also minimize their probability of starvation, where starvation is defined as some minimum food requirement that they need to achieve by the end of a day’s (or night’s) foraging^1^,^2^. Depending on their energy reserves, individuals might prefer variable food sources that have an occasional high reward over lower rewarding (but constant) food resources. In short, if an animal is in danger of starvation it may prefer high reward but risky foraging sites over low-reward but safer, more constant, foraging sites. Reciprocally, if it is well nourished, it may favour reliable but low-reward sites over variable high reward sites. Risk-sensitivity in foraging strategy has been demonstrated empirically across a broad range of animals including birds^1^,^3^,^4^,^5^,^6^, shrews^7^, primates^8^ and social insects^9^,^10^,^11^,^12^,^13^.

To determine if animals are sensitive to resource variability, researchers typically assess animal preference by offering individuals a choice between either a variable reward or a constant reward^14^,^15^. Alternatively, researchers have investigated how animals perceive the relative value of the different attributes of a food source such as caloric content versus ease of collecting or imbibing food^16^,^17^,^18^,^19^. Here we take a different approach by studying the risk mitigation strategy employed by honey bees as they travel between their nest and a food source. When a honey bee forager leaves her nest, she carries with her a small amount of food in her crop to use as fuel on the outward journey^20^,^21^,^22^. In *A. mellifera* the fuel is solicited from hive bees just prior to departure^20^. The further away the food source, and the less familiar the forager is with its location, the more fuel she will take with her^21^,^22^,^23^. A honey bee forager that carries insufficient fuel on her outward journey and does not locate a food source of sufficient quality risks starvation in the field. Therefore the amount of ‘fuel’ taken by a foraging bee on her outward journey may be an objective measure of her perception of the riskiness of the foraging trip she is embarking on. An individual bee that perceives her foraging trip as being risky should carry more fuel as she perceives that the energy budget of the trip may place her at risk of starvation before she can return to the nest.

Here we examine the fuel loads carried by honey bees on their outward journeys to a familiar food source that provided either a constant reward or a variable reward. A demonstration that individual foragers increase their fuel load when foraging on a variable food source relative to the fuel they carry when departing for a site providing constant reward would show that honey bees alter their behavior according to their perceptions of the riskiness of resource variability.

## Results

**Figure 1:**
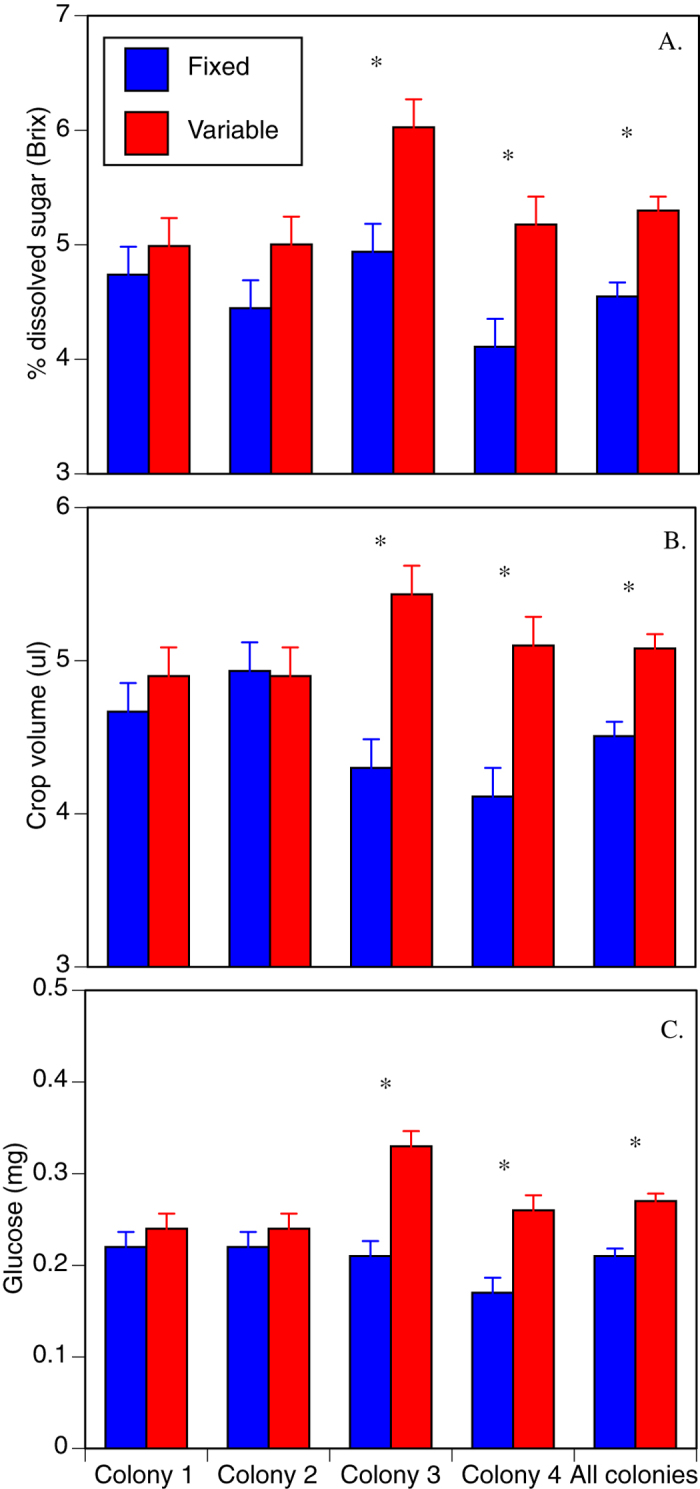
Changes in fuel load carried by Eastern hive bees foraging on a 1.2 km-distant feeder providing either constant (1.5 M sucrose solution) or variable (0.5, 1.5, 2.5 M) rewards. (**A**) % dissolved sucrose (Brix) measured by a refractometer. (**B**) Volume of liquid in the crop (μl). (**C**) The glucose content of the fuel load. Error bars are standard errors of the mean.

Whether or not the sucrose concentration available at a familiar feeding station was constant, or varied over time, had a highly significant (*P* < 0.001) effect on the volume, sugar concentration and amount of glucose-equivalent sugar carried by departing foragers (Table 1, Fig. 1). All four colonies showed the same trend towards increased dissolved sugar and total glucose equivalents, and three of four colonies for increased crop volume, when foraging on a variable food source (Fig. 1). Two of the four colonies showed the effect very strongly for all three measures (Fig. 1), leading to a highly significant effect of treatment on these variables overall, and to significant colony by treatment interactions for volume and amount of glucose (Table 1). Our bees carried a mean of 0.27 ± s.e. 0.008 mg of glucose-equivalent sugar in a 5.08 ± 0.09 μl volume on their outward journeys when the feeder provided variable sucrose concentration, but only 0.21 ± 0.008 mg in 4.5 ± 0.09 μl when it was uniform. Thus on average, foragers carried 0.57 ± 0.13 μl or 12.7% greater volume of fuel when foraging on a variable source than when forging on a consistent source, though this was variable between colonies (range 0.3–1.3 μl) (Fig. 1A). The fuel load was also significantly more concentrated when bees were foraging on a variable resource (Table 1). The combined change in volume and concentration meant that when bees were foraging on a variable resource they carried 0.06 ± 0.01 mg or 30.2% more glucose-equivalent sugar than when foraging on a constant resource.

**Figure 2:**
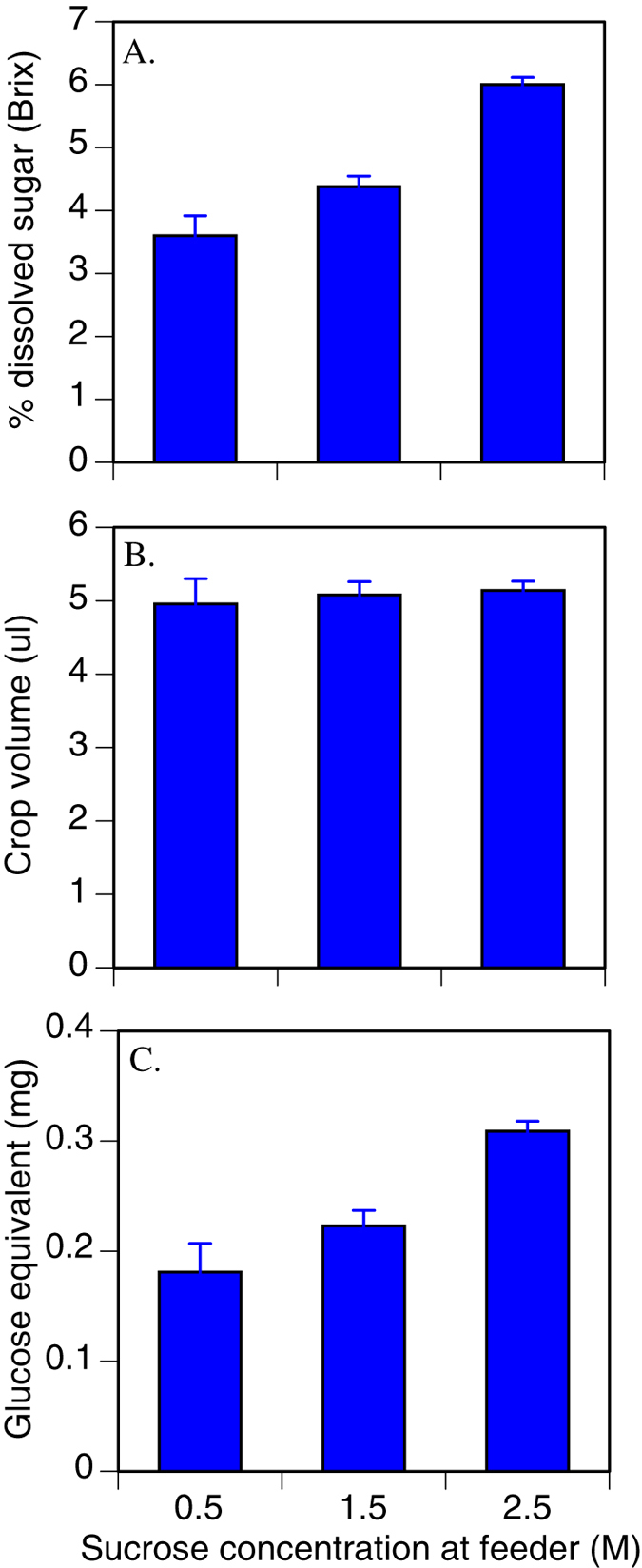
The influence of sucrose concentration of the forager’s last trip to the feeder. (**A**) % dissolved sucrose (Brix) of the crop contents increased with sucrose concentration at the feeder on the forager’s last trip to feeder (Generalized Linear Model, Wald χ^2^ = 0105.2, d.f. = 2, *P* < 0.001). Similarly, glucose equivalent (**C**) was influenced by the sucrose concentration on the forager’s last trip to feeder (χ^2^* *= 43.9, d.f.* *= 2, *P* < 0.001). However there was no effect on the volume (**B**), which was constant irrespective of the sucrose concentration at the feeder on the last trip (χ^2^* *= 0.32, *P *= 0.85). Error bars are standard errors of the mean.

The finding that crop concentration and volume were influenced by feeder variability could indicate either that bees were responding to feeder variability *per se*, or alternatively, that bees were altering their behaviour according to the quality of the most recent feeder they experienced. To tease these two alternatives apart, we analyzed the influence of previous feeder quality on crop contents sugar concentration and volume. On the days when the feeders offered variable rewards, the sugar concentration, and the total glucose-equivalent of the crop contents was significantly associated with the sucrose content of the syrup offered at the feeder on each bee’s most recent trip (Fig. 2). This may have arisen because some residual sucrose was retained from the previous trip, and because the higher the sucrose concentration at the feeder on the most recent trip, the more likely the bee was to turn around quickly and be captured. Over all colonies we caught 10 bees after a trip when the feeder contained 0.5 M sucrose, 36 after 1.5 M, and 74 after 2.5 M. Thus the probability of capture was significantly associated with the sucrose concentration at the feeder on the most recent foraging trip (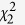
= 51.4, *P* < 0.001, null hypothesis that the number of workers captured after each feeder concentration should be equal). This probability was similar across all four colonies (colony by concentration contingency table of forager counts, Fisher’s exact test, *P *= 0.79). The concentration of the feeder encountered on the previous trip had no discernable impact on the volume of syrup carried by departing bees (Fig. 2).

No precipitation was recorded on any of the experimental days. The maximum temperature on the days of fixed rewards was 25.7 C (s.e.* *= 0.7, range 25.2-26.2) and on days of variable rewards it was 25.8 C (s.e.* *= 0.5, range 25.5–26.2), which are not statistically different (*P *= 0.54, paired *t*-test).

## Discussion

*A. cerana* carried 12.7% greater volume of fuel when a feeder offered variable rewards relative to that which they carried when a feeder offers constant reward. This suggests that bees adjust their fuel load depending on their perceived risk of not finding an adequately profitable food source, and thereby compensate for their aversion to variability in reward.

The exact cause of the behavioural response to changes in food reward variability is arguable. Two hypotheses are plausible. First, bees may respond to variability itself, perhaps by hedging against a prolonged wait time for the feeder to become more profitable, or the need to return home with low quality sugar. This hypothesis is compatible with the evidence that individual bees prefer constant rewards over variable rewards^14^,^15^,^24^. Alternatively, bees may not respond to the variability itself, but to the possibility of a low (0.5 M) reward. That is, there may be a threshold effect, and the bees may carry fuel appropriate for the lowest possible reward that they may encounter. However the lack of association between crop volume and sucrose concentration at the feeder on the most recent foraging trip is the opposite to what might be predicted under this hypothesis: the bees carried a similar volume of fuel if they had foraged on 0.5 M or 2.5 M syrup on their most recent trip (Fig. 2). We therefore suggest that bees respond to the variability itself, but still cannot exclude the possibility that they respond to the minimum concentration that they have encountered.

We found a significant increase in the concentration of crop contents when bees were foraging on a variable resource relative to a constant resource. Departing bees foraging on a feeder offering variable sucrose concentrations had crop contents with 16.2% greater concentration of dissolved sugar and 30% total glucose relative to those foraging on a constant source (because of the greater volume and concentration). Importantly, the crop contents were far more dilute than that available at the feeder, so the majority of the crop contents of exiting foragers was not residual sucrose from the most recent foraging trip. In *A. mellifera* the fuel load is thought to be acquired by trophallaxis from other bees just prior to departure^20^, and we assume that *A. cerana* do the same. Nonetheless, the strong association between the sugar content of the crop and the concentration of sucrose on the last foraging trip may arise because bees retain some liquid from their last foraging trip in their crop. Further, we were significantly more likely to catch bees after they had foraged on the 2.5 M solution than after they had foraged on a 1.5 M or 0.5 M solution. The combination of increased likelihood of capture, and retention of some liquid from the previous trip could have led to an increase in sugar concentration in the bees foraging on a feeder with variable rewards.

To examine this phenomenon further, we compared the amount of dissolved sucrose in the crops of workers that had last foraged on a 1.5 M sucrose source between the variable and constant treatments. Workers from the variable treatment had crop contents with slightly less dissolved sugar (4.4 ± 0.24 Brix, n* *= 36) than workers foraging on constant sources (5.05 ± 0.13 Brix, n* *= 120). The difference is significant (Generalized Linear Model, Wald χ^2^* *= 6.11, *P *= 0.013). This analysis therefore does not provide evidence that bees foraging at a variable resource increase the sugar content of their fuel load, because it is in the opposite direction to what is expected under this hypothesis. Most likely the effect arises from the low sample size in the variable treatment.

We conclude that foragers adjust volume of their fuel load in response to variable food sources. While they may also increase the sucrose content of the fuel load we cannot exclude the possibility that it is an artifact of residual sugar from the previous foraging trip. We note that because foragers obtain fuel for their outward journey via trophallaxis, it is difficult to envisage how they could adjust the sucrose content of their fuel^20^.

There were significant effects of colony on the sugar concentration of fuel loads, but not the volume. Colony-level effects may have arisen for several reasons. First, we studied colonies sequentially over a period of one month. At both the individual and colony level, *A mellifera*, adaptively tune their perception of food value in response to the quality of food being brought to the colony, and the amount of food that is stored in the colony^25^. This means that if the (limited) forage available in the field changed between colonies during our experiments, then this variability may have contributed to the significant colony-level effects^26^,^27^,^28^. Importantly, all colonies responded to variance in reward in a similar direction with respect to dissolved sugar and total glucose (Fig. 1), although colonies 3 and 4 responded more strongly than did colonies 1 and 2. All but colony 2 responded in the same direction for volume (Fig. 1). Second, weather conditions might affect forager perceptions of risk. However the weather was stable and optimal for foraging during our studies, with no significant differences in maximum temperature between days of uniform and variable sucrose concentration at the feeder. Therefore temperature fluctuations are unlikely to explain inter-colony heterogeneity.

*Apis mellifera* are less likely to advertise a food source to nest mates by dancing if the source provides variable rewards^24^. This provides additional evidence of aversion to variability in rewards^13^,^14^,^15^. It supports our hypothesis that honey bee foragers can recognize that a forage source provides variable rewards, and take action to mitigate the risk of starvation on a foraging trip. At an individual level, foragers respond to variability by either choosing another less variable resource^14^,^15^,^24^. or by increasing the crop volume on the outward journey (this study). At the collective level, foragers respond by reduced frequency of recruitment dances^2^, reducing risks to recruits.

In conclusion, previous studies have shown that honey bee foragers prefer constant rewards over variable rewards^14^,^15^. Our study shows that when *A. cerana* foragers are faced with variable rewards at a feeding site, they mitigate the risk by increasing the volume, and possibly the concentration of the fuel load they carry. These findings support an earlier study that showed that *A. mellifera* reduce the volume of fuel they carry as they become more familiar with a foraging site^21^. Finally, fuel load volume seems to provide a direct measure of a forager’s perception of the riskiness of a foraging trip, and may provide a novel experimental tool for determining how foragers rank riskiness.

## Methods

### Training of bees

Experiments were conducted in August 2014 at Yunnan Agricultural University, Kunming, China (22°42’30 N, 100°56’01 E, 1890 m altitude) during a period of constant, warm temperatures and floral dearth, which facilitates feeder training of bees.

We sequentially used four colonies of the Eastern honey bee, *A. cerana*, each with four frames of bees and brood. For each experimental colony, we captured about 60 foragers at the colony entrance and placed them into individual opaque tubes. We then released the bees one at a time at a feeder placed 1,200 m from the focal colony^29^. Our experience shows that this is the best way to train *A. cerana* foragers to a distant feeder, and more efficient than slowly luring trained bees away from their colony by moving a feeder away as is the standard training protocol for *A. mellifera*. We deemed 1,200 m to be necessary so that the bees would require considerable fuel for their outward journey. (*A. mellifera* carries about 2.8 μl of fuel food on an outward journey of 1 km^21^). The feeder, which was scented with citrol rested on a blue card, consisted of an inverted 70 mL vial with 18 slots drilled around the lid^30^. During the training phase the feeder contained a 2 M sucrose solution. If a released bee began to imbibe food, we marked her with a numbered bee tag (Opalith-Zeichenplättchen) affixed to the thorax with shellac. We continued until we had >30 individually marked bees foraging at the feeder.

Throughout the training, if an unmarked bee arrived at the feeder it was uniquely marked. An observer at the feeder then called an observer at the experimental colony on a walkie-talkie. If the newly-marked bee was not observed returning to the colony she was killed on her return to the feeder. In this way we prevented bees from non-experimental colonies from foraging at our feeder, and ensured that all bees from the experimental colony that were foraging at the feeder were marked.

### Manipulation of variability in rewards

After >30 bees were regularly foraging on the training feeder we initiated the first treatment in which we changed the contents of the feeder every 15 min in a random sequence of 0.5, 1.5 and 2.5 M sucrose solution. We chose these concentrations because it is our experience that at our location, foragers find 2.5 M highly attractive, 0.5 M unattractive and 1.5 M intermediate^17^,^31^. Bees foraged on the manipulated feeder for one full day and for the morning of the next day. We then captured 30 marked bees as they exited the colony after returning from the feeder. The person catching bees as they exited the colony was unaware of the sucrose concentration at the feeder on the forager’s last trip, but we used the notes of the observer at the feeder and the bee’s individual marks to determine what sugar concentration the departing bee had last imbibed.

On the following day we initiated the second treatment. The average quality was the same when the feeder was providing variable and constant rewards: 1.5 M. We trained 30 naïve (unmarked) bees to the feeder using the method described above. After training, the contents of the feeder was held constant at 1.5 M for one full day, for the morning of the next day, and throughout the foraging period when we again captured 30 departing foragers. It is important to note that the second 30 bees had never experienced the variable feeder and were an independent sample. The crop contents of departing foragers decreases with the bee’s experience with the feeding site, and is minimized after one hour of foraging or greater than five trips^21^. Our bees had all foraged on the feeder for more than 24 hours, and should therefore have stabilized on the minimum fuel load appropriate for the journey^21^.

### Crop contents

To measure the crop contents of the captured bees we pinned them to a dissection dish through the thorax. We then pulled the abdomen from the thorax using forceps to expose the crop^21^. The volume of the crop contents was then quantified by drawing the contents into a 50-μL Hamilton syringe (Reno, USA)^21^. After recording the volume of the fuel load by reading the length of the column of liquid in the syringe we ejected the contents onto the lens of a refractometer (WYA-2S, Yidianwuguang, Shanghai) and measured the concentration of dissolved sugars.

To estimate the weight of glucose equivalents in milligrams in the crop contents we first determined the refractometer reading for 0.5, 1.0, 1.5 and 2.5 M glucose solutions. From these readings we obtained a standard curve to convert refractometer readings to the molar concentration of dissolved glucose. We then multiplied the volume of the crop contents by the molarity. Note that because sucrose is comprised of one molecule of glucose and one of fructose, sucrose solutions measure approximately twice the sugar content of glucose solutions on a refractomenter for the same molarity. In other words, for the same refractometer reading, the glucose content of a solution is similar regardless of the proportion of the glucose molecules that are present as components of other, more complex sugars.

### Weather during the experiment

Bee foraging is influenced by weather conditions. To verify that weather conditions were stable during our experiments, data were obtained from the University’s weather station located 400 m from the experimental colonies.

### Statistical analysis

We used Generalized Linear Models to assess the effects of two predictor variables: colony and treatment (variable rewards or fixed rewards) or colony and sucrose concentration at the feeder on the last foraging trip. The dependent variables for both models were: % dissolved sugar (Brix) as measured by the refractometer, volume, and the estimated weight of glucose carried in the crop of departing foragers. We specified a normal distribution and a linear link function. We compared the least square means based on least significant differences.

## Additional Information

**How to cite this article**: Tan, K. *et al.* Individual honey bee (*Apis cerana*) foragers adjust their fuel load to match variability in forage reward. *Sci. Rep.* **5**, 16418; doi: 10.1038/srep16418 (2015).

## Figures and Tables

**Table 1:** The effects of treatment (variable or constant sucrose rewards at the feeder) and colony (1–4) on the crop contents of departing foragers.

Source	% dissolved sugar			Volume			Glucose mass		
	Wald χ^2^	d.f.	*P*	Wald χ^2^	d.f.	*P*	Wald χ^2^	d.f.	*P*
Overall fit of the model	34.74	7	<0.001	33.10	7	<0.001	49.04	7	<0.001
Colony (C)	14.7	3	0.002	3.0	3	0.4	12.7	3	0.005
Treatment (T)	18.5	1	<0.001	18.9	1	<0.001	29.3	1	<0.001
CxT	4.2	3	0.24	13.6	3	0.003	12.3	3	0.006

## References

[b1] Caraco T., Martindale S. & Whittam T. S. An empirical demonstration of risk-sensitive foraging preferences. Anim. Behav. 28, 820–830, 10.1016/S0003-3472(80)80142-4 (1980).

[b2] Stephens D. W. The logic of risk-sensitive foraging preferences. Anim. Behav. 29, 628–629, 10.1016/S0003-3472(81)80128-5 (1981).

[b3] Barkan C. P. L. A field test of risk-sensitive foraging in black-capped chickadees (*Parus atricapillus)*. Ecology 71, 391–400, 10.2307/1940276 (1990).

[b4] Hurly T. A. & Oseen M. D. Context-dependent, risk-sensitive foraging preferences in wild rufous hummingbirds. Anim. Behav. 58, 59–66, 10.1006/anbe.1999.1130 (1999). 10413541

[b5] Bautista L. M., Martin B., Martinez L. & Mayo C. Risk sensitive foraging in coal tits. Behaviour 138, 69–83, 10.1163/156853901750077790 (2000).

[b6] Bateson M. Context-dependent foraging choices in risk-sensitive starlings. Anim. Behav. 64, 251–260, 10.1006/abe.2002.3959 (2002).

[b7] Barnard C. J. & Brown C. A. J. Risk-sensitive foraging in common shrews (*Sorex araneus* L.). Behav. Ecol. Sociobiol. 16, 161–164, 10.2307/4599760 (1985).

[b8] MacLean E. L., Mandalaywala T. M. & Brannon E. M. Variance-sensitive choice in lemurs: constancy trumps quantity. Anim Cognit 15, 15–25, 10.1007/s10071-011-0425-2 (2011). 21670948 PMC3645319

[b9] Real L. A. Nectar availability and bee-foraging on *Ipomoea* (Convolvulaceae). Biotropica 13, 95–109 (1981).

[b10] Cartar R. V. A test of risk-sensitive foraging in wild bumble bees. Ecology 72, 888–895, 10.2307/1940590 (1991).

[b11] Cartar R. V. & Dill L. M. Why are bumble bees risk-sensitive foragers? Behav. Ecol. Sociobiol. 26, 121–127, 10.1007/BF00171581 (1990).

[b12] Thiyagesan K., Varadharajan M. & Abirami S. Studies on risk-sensitive foraging in the Indian honey bee (*Apis cerana indica*). J. Apic. Res. 40, 16–20 (2001).

[b13] Mayack C. & Naug D. A changing but not an absolute energy budget dictates risk-sensitive behaviour in the honeybee. Anim. Behav. 82, 595–600, 10.1016/j.anbehav.2011.06.022 (2011).

[b14] Shafir S., Wiegmann D. D., Smith B. H. & Real L. A. Risk-sensitive foraging: choice behaviour of honeybees in response to variability in volume of reward. Anim. Behav. 57, 1055–1061, 10.1006/anbe.1998.1078 (1999). 10328791

[b15] Shafir S. Risk-sensitive foraging: The effect of relative variability. Oikos 88, 663–669, 10.2307/3546957 (2000).

[b16] Nardone E., Dey T. & Kevan P. G. The effect of sugar solution type, sugar concentration and viscosity on the imbibition and energy intake rate of bumblebees. J. Ins. Physiol. 59, 919–933, 10.1016/j.jinsphys.2013.06.007 (2013). 23831183

[b17] Tan K. *et al.* Preferences and tradeoffs in nectar temperature and nectar concentration in the Asian hive bee *Apis cerana*. Behav. Ecol. Sociobiol. 68, 13–20, 10.1007/s00265-013-1617-3 (2014).

[b18] Nicolson S. W., de Veer L., Köhler A. & Pirk C. W. W. Honeybees prefer warmer nectar and less viscous nectar regardless of sugar concentration. Proceedings of the Royal Society B 280, 20131597, 10.1098/rsb.2013.1597 (2013). 23902913 PMC3735266

[b19] Shafir S. & Yehonatan L. Comparative evaluations of reward dimensions in honey bees: evidence from two-alternative forced choice proboscis-extension conditioning. Anim Cognit 17, 633–644, 10.1007/s10071-013-0694-z (2014). 24121898

[b20] Beutler R. Zeit und Raum im Leben der Sammelbiene. Naturwissenschaften 37, 102–105 (1950).

[b21] Harano K.-I., Mitshuhata-Asai A., Konishi T., Suzuki T. & Sasaki K. Honeybee foragers adjust crop contents before leaving the hive. Behav. Ecol. Sociobiol. 67, 1169–1178, 10.1007/s0026-5013-1542-5 (2013).

[b22] Harano K., Mitsuhata-Asai A. & Sasaki M. Honey loading for pollen collection: regulation of crop content in honeybee pollen foragers on leaving hive. Naturwissenschaften 101, 595–598, 10.1007/s00114-014-1185-z (2014). 24925356

[b23] Beutler R. Time and distance in the life if the foraging bee. Bee Wld. 32, 25–27 (1951).

[b24] Seefeldt S. & De Marco R. J. The response of the honeybee dance to uncertain rewards. J. Exp. Biol. 211, 3392–3400, 10.1242/jeb.017624 (2008). 18931312

[b25] Seeley T. D. Social foraging in honey bees: how nectar foragers assess their colony nutritional status. Behav. Ecol. Sociobiol. 24, 181–199 (1989).

[b26] Seeley T. D. Social foraging by honey bees: How colonies allocate foragers among patches of flowers. Behav. Ecol. Sociobiol. 19, 343–354, 10.1007/BF00295707 (1986).

[b27] Seeley T. D., Camazine S. & Sneyd J. Collective decision-making in honey bees: how colonies choose among nectar sources. Behav. Ecol. Sociobiol. 28, 277–290, 10.1007/BF00175101 (1991).

[b28] Seeley T. D. Honey bee foragers as sensory units of their colonies. Behav. Ecol. Sociobiol. 34, 51–62, 10.1007/BF00175458 (1994).

[b29] Núñez J. A. Honeybee foraging strategies at a food source in relation to its distance from the hive an the rate of sugar flow. J. Apic. Res. 21, 130–150 (1982).

[b30] Frisch K. V. The dance language and orientation of bees. (Harvard University Press, 1967).

[b31] Tan K. *et al.* Phantom alternatives influence food preferences in the eastern honeynee *Apis cerana*. J. Anim. Ecol. 84, 509–517, 10.1111/1365-2656.12288 (2014). 25251672

